# Assessment of knowledge, attitude, and practice regarding diabetes among patients with type 2 diabetes in Hunan province, China: a cross-sectional study

**DOI:** 10.3389/frhs.2026.1791049

**Published:** 2026-03-24

**Authors:** Guiyan Chen, Ning Gao, Jianwei Huang, Linhua Pi

**Affiliations:** 1 Department of Endocrinology and Metabolism, The Second Affiliated Hospital of Guilin Medical University, Guilin, Guangxi, China; 2 Department of Respiratory and Critical Care Medicine, The Second Affiliated Hospital of Guilin Medical University, Guilin, China; 3 Guangxi Health Commission Key Laboratory of Glucose and Lipid Metabolism Disorders, The Second Affiliated Hospital of Guilin Medical University, Guilin, Guangxi, China; 4 Guangxi Clinical Research Center for Diabetes and Metabolic Diseases, The Second Affiliated Hospital of Guilin Medical University, Guilin, Guangxi, China

**Keywords:** China, cross-sectional study, diabetes, diabetic patients, KAP

## Abstract

**Background:**

Type 2 diabetes mellitus (T2DM) has become a public health crisis in China, particularly in rural areas, leading to significant impairment of quality of life and premature death. However, little is known about the level of knowledge, attitude, and practice (KAP) required for diabetic patients for effective management and prevention of complications. This study aimed to assess the KAP level of patients with T2DM and identify the associated factors in Central China.

**Methods:**

A cross-sectional study was conducted using self-administered KAP questionnaires among patients with T2DM recruited from 12 township health centers. KAP levels were determined by calculating the scores, and the association between respondents' characteristics and KAP outcomes was evaluated using chi-square tests and Mann–Whitney *U*-tests, as appropriate.

**Results:**

In total, 259 diabetic patients completed the survey. Overall, despite adequate knowledge and positive attitudes toward T2DM, there were substantial gaps in practices. The respondents scored 13.09 out of 17points on the knowledge subscales, 3.88 out of 4 on the attitude subscales, and 4.96 out of 12 on the practice subscales. Educational attainment, health insurance type, diabetic complications, and current medical treatment pattern were significant predictors of knowledge. Educational attainment, health insurance type, diabetic complications, and comorbidities were significant predictors of attitudes. Educational attainment and marital status affected respondents' practices significantly.

**Conclusions:**

Despite adequate knowledge and positive attitudes toward T2DM, there were substantial gaps in diabetic practices. These findings highlight the urgent need for action from relevant health authorities and policymakers to improve diabetic practices among Chinese patients with T2DM in rural China.

## Introduction

Diabetes is a significant rapidly growing global public health crisis, posing a major threat to public health worldwide. At present, nearly 537 million people aged 20–70 years are living with diabetes, and this number is projected to increase to 643 million by 2030 (1). Due to its rapid economic transition and population aging, China has witnessed the most dramatic rise in diabetes prevalence. The latest epidemiological study indicates that approximately 12.8% of the Chinese population has diabetes, representing up to 129.8 million citizens (2). Type 2 diabetes accounts for nearly 90% of all cases. However, in China, many cases of diabetes are not adequately managed. A recent study revealed that the proportion of people who achieved optimal control of hemoglobin A1c (HbA1c), blood pressure, and low-density lipoprotein cholesterol was low in China—64.1%, 22.2%, and 23.9%, respectively—and only 4.4% achieved all three target (3). These findings demonstrate substantial gaps in diabetes management and highlight the urgent need for actions to improve diabetes management.

Historically, diabetes was deemed more prevalent among the urban population as compared with the rural population in China (4, 5). However, recent studies found no significant differences in the prevalence of diabetes between urban and rural residents (2). Meanwhile, awareness, treatment, and control rates of diabetes in rural populations remain lower. China has a large rural population with limited medical resources; therefore, a large number of people are at risk of developing diabetes, and without the implementation of effective preventive measures in rural areas, there is increased risk of diabetic complications as well.

Effective diabetes management require self-care behaviors and lifestyle modifications, including adherence to diabetic medication, a balanced diet, regular exercise, smoking and alcohol cessation, and other behaviors that are influenced by an individual's health literacy as well as their knowledge, attitudes, and practices. Studies have indicated that low health literacy was associated with poor knowledge and worse glycemic control (6, 7). Moreover, knowledge, attitude, and practice (KAP) regarding diabetes play a critical role during the management of the disease. Knowledge regarding diabetes can influence medication adherence (8), while attitude affects adherence to diabetic foot care (9), exercise (10), and the prevention of complications (11). Several studies using KAP-based questionnaires have demonstrated that many participants have poor knowledge, negative attitudes, and inadequate practices (12–14). For example, a study conducted in China showed that citizens in Shanghai possessed adequate knowledge and high levels of perceived attitudes regarding diabetes, but the practices of participants remained poor (15). There is a paucity of studies assessing KAP regarding diabetes among rural diabetic patients in China. Rural populations are often underserved due to health resource limitation and geographic isolation (16). A study suggested that the rural‒urban care gap for patients with chronic disease still persists in China, with rural residents having less access to diabetes care due to inadequate infrastructure (17). To the best of our knowledge, no study has assessed KAP among diabetic patients in rural areas in Central China. This study therefore aimed to evaluate the KAP of diabetic patients in rural areas of Central China and analyze relevant influencing factors. The findings are expected to provide better insight into strategies to improve the management of type 2 diabetes mellitus (T2DM) among these socioeconomically disadvantaged populations.

## Methods

### Study design

This was a community-based cross-sectional study at 12 township health centers in Pingjiang City, Hunan Province. All patients with T2DM receiving diabetes care in the outpatient department in the 12 health centers were eligible for inclusion in this study.

### Participants

Inclusion criteria were as follows: (1) age 18 years or older with ability to understand or communicate; (2) diagnosis of T2DM more than 1 year; and (3) provision of informed consent to participant in this study. Exclusion criteria included patients with type 1 diabetes, cognitive impairment, or extreme disease conditions (such as malignant tumor or psychiatric illness).

### Study sample

A simple random sampling method was used to select 400 type 2 diabetic patients who were managed by the 12 health centers. Questionnaire were distributed through an online platform in China, which provided functions equivalent to Amazon Mechanical Turk, enabling online questionnaire surveys, examinations, assessments, and voting. We shared the survey link through text messages or WeChat; participants willing to finish the survey could click the link and submit their results automatically when they finished. At a significance level of 95% (*z*), given the prevalence of adequate levels of knowledge about diabetes, positive attitude, and practice of 50% (*p*), with a 5% error margin (*d*), the minimum computed sample size was 218. Accounting for a 10% contingency, the resulting minimum sample size was 242.

### Ethical considerations

The study adhered to the principles of the Declaration of Helsinki. Ethics approval was obtained from the Ethics Committee of the Second Xiangya Hospital of Central South University (Number: No. 006, 2022).

### The questionnaire

The questionnaire was adapted from relevant literature (18–20) and validated by two endocrinologists and one primary care physician. A pretest was conducted among 15 patients to test the reliability and improve the clarity of the questionnaire.

The questionnaire consisted of four sections. The first part of the questionnaire focused on patients' demographic information that included gender, age, level of education, occupation, marital status, household income, diabetic duration, diabetic complications, comorbidities, and current treatments. The second part consisted of 18 questions on the participants' diabetes-related knowledge, including T2DM definition, risk factors, long-term damage of T2DM, diet and exercise for diabetic patients, indications for insulin use, signs and treatment of hypoglycemia, and the target index of fasting plasma glucose and blood pressure. The third part consisted of four questions on patients' attitude toward diabetes, which included the importance of education, diet and exercise, therapy adherence, and regular self-blood glucose monitoring for management of T2DM. The final part measured the usual behaviors related to diabetes practice using 12 questions on diet, exercise, regular self-blood glucose monitoring, blood pressure measurement, medication, foot care, and actively seeking medical services for periodically blood lipid, renal function, and eye examinations in the last year.

Questions related to knowledge and practice were evaluated using a two-point scale (1 point for correct answer, 0 for false or not sure). Questions related to attitude were evaluated using a five-point Likert scale (1 for strongly agree and agree, 0 for uncertain, disagree, and strongly disagree). Finally, questions related to practice were also evaluated using a five-point Likert scale (1 for always and frequently, 0 for sometimes, seldom, and never). The total scores ranged from 0 to 18, 0 to 4, and 0 to 12 for knowledge, attitude, and practice, respectively. Higher scores indicated better knowledge, attitude, and practice. The reliability of the questionnaire was assessed through the internal consistency method after it was completed by 15 patients. Cronbach's alpha was 0.823 for the total questionnaire and 0.805, 0.847, and 0.837 for the knowledge, attitude, and practice subscales, respectively.

### Statistical analyses

Data were analyzed using SPSS software version 25.0 (IBM Corporation, Chicago, IL, USA). Participants' demographic data and KAP scores were reported using descriptive statistics. The normal distribution of quantitative variables was examined using the Kolmogorov‒Smirnov test before the data analysis. Normality testing showed that the duration of diabetes in the study did not follow a normal distribution (*p* < 0.05); therefore, it was presented as the median (interquartile range). Associations between participants’ characteristics and KAP scores were evaluated by chi-square tests or Mann‒Whitney *U*-tests, as appropriate. A *p* value < 0.05 was considered statistically significant.

## Results

### Patient demographic and clinical characteristics

This study was conducted between 20 April and 30 July 2023. Out of 400 patients with T2DM approached, 259 agreed to participate in our study (response rate of 64.8%). The majority of our diabetic respondents were female (144, 55.6%), aged 40–59 years (157, 60.6%), married (236, 91.1%), had a primary and secondary school certificate (137, 52.9%), and possessed health insurance. The average duration of diabetes mellitus was 5.1 years, with most of participants having acquired it within the past 5 years (156, 60.2%); 20.1% had diabetic complications and 54.1% had comorbidities. Among the surveyed diabetic patients, 28.2% were treated with oral hypoglycemic medication, 8.1% with insulin, 10.8% with oral hypoglycemic medication and insulin, and 52.9% were untreated (Table 1).

**Table 1:** Demographic and clinical characteristics of the study participants (*N* = 259).

Variable	*n* (%)
Gender	
Female	144 (55.6)
Male	115 (44.4)
Age, years	53.2 ± 11.7
<40	27 (10.4)
40–59	157 (60.6)
≥60	75 (29.0)
Marital status	
Single	11 (4.2)
Married	236 (91.2)
Divorced/widowed	12 (4.6)
Educational attainment	
Illiterate	10 (3.9)
Primary and secondary school	137 (52.9)
High school	49 (18.9)
College and higher degrees	63 (24.3)
Health insurance	
No	11 (4.2)
Yes	248 (95.8)
Duration of diabetes mellitus (years)	5.2 (2.8–8.6)
<5	156 (60.2)
5–9	79 (30.5)
≥10	24 (9.3)
Diabetic complications	
No	207 (79.9)
Yes	52 (20.1)
Comorbidities	
No	119 (45.9)
Yes	140 (54.1)
Current medical treatment	
Without medicine	137 (52.9)
Oral medicine	73 (28.2)
Insulin	21 (8.1)
Oral medicine + insulin	28 (10.8)

Data are expressed as mean ± standard deviation, median (Q1–Q3) or *n* (%).

### Patients' diabetes-related knowledge

Participants responded correctly to questions relating to the definition of T2DM (72.6%), identified overweight or obese people (70.3%), and knew that a family history of diabetes is a risk factor of diabetes. Regarding symptoms of diabetes, 70.3% were aware that polyuria, thirst, hunger, and weight loss are classic symptoms of T2DM. Diabetic complications included stroke, vision loss or blindness, renal failure, and amputation of limbs, which were identified by 67.6%, 87.3%, 84.2%, and 83.0% of surveyed participants, respectively. Among participants, 83.0% and 79.2% recognized daily energy restriction and cooking oil restriction, respectively, as important components of diabetic management. Regarding exercise, 81.5% believed that it was helpful to consult a doctor about precautions and contraindications before making an exercise plan, and 79.5% were aware of the minimum recommended duration of exercise. In terms of treatment targets, 67.2% and 74.5% knew the expected goals for blood sugar and blood pressure, respectively. Moreover, 70.7% understood that insulin therapy should be started immediately when oral medication is not effective, 84.2% could identify the signs of hypoglycemia, and 77.6% could treat hypoglycemia correctly (Table 2).

**Table 2:** Description of patients’ knowledge scores in detail.

Item	Correct choice *n* (%)	Score (0–1) mean ± SD
K1. The definition of T2DM	188 (72.6)	0.73 ± 0.45
K2. Overweight or obese are risk factors of T2DM	182 (70.3)	0.70 ± 0.46
K3. The family history of diabetes is the risk factors of T2DM	197 (76.1)	0.76 ± 0.43
K4. The classic symptoms of T2DM	182 (70.3)	0.70 ± 0.46
K5. Poorly controlled blood glucose may lead to stroke	175 (67.6)	0.78 ± 0.47
K6. Poorly controlled blood glucose may lead to vision loss or blindness	226 (87.3)	0.87 ± 0.33
K7. Poorly controlled blood glucose may lead to renal failure	218 (84.2)	0.84 ± 0.37
K8. Poorly controlled blood glucose may lead to amputation of limbs	215 (83.0)	0.83 ± 0.38
K9. Control of the total daily energy intake is key for diabetic patients	215 (83.0)	0.83 ± 0.38
K10. The recommend daily intake of oil for patients with T2DM	205 (79.2)	0.79 ± 0.41
K11. Patients with diabetes should consult a doctor about the precautions and contraindications of exercise before making an exercise plan	211 (81.5)	0.81 ± 0.39
K12. The recommended exercise goal of patients with T2DM	206 (79.5)	0.80 ± 0.40
K13. The recommended control target for fasting blood glucose for patients with T2DM	174 (7.2)	0.67 ± 0.47
K14. The recommended control target of hypertension for patients with T2DM	193 (74.5)	0.75 ± 0.44
K15. The optimal time to initiate insulin when oral medication is not effective	183 (70.7)	0.71 ± 0.45
K16. The sign of hypoglycemia	218 (84.2)	0.84 ± 0.37
K17. The best way to relieve hypoglycemia	201 (77.6)	0.78 ± 0.42
Total	3,389 (76.7)	13.09 ± 0.42

### Patients' diabetes-related attitude

The attitudes of the diabetic patients were assessed using four questions. The majority of respondents strongly believed that T2DM affects nearly every part of a patient’s life (98.1%) and that diabetic complications can be prevented through blood glucose control (97.7%). Most participants also considered regular self-monitoring of blood glucose (95.0%) and adherence to diet and exercise recommendations (97.3%) as vital for diabetes management (Table 3).

**Table 3:** Description of patients’ attitude scores in detail.

Item	Positive attitude *n* (%)	Score (0–1) mean ± SD
A1. T2D May affect almost every part of a diabetic person's life.	254 (98.1)	0.98 ± 0.14
A2. DM complications may be prevented if blood glucose level is well controlled.	253 (97.7)	0.98 ± 0.15
A3. Self-monitoring of blood glucose is important for of T2DM management.	246 (95.0)	0.96 ± 0.25
A4. Diet and exercise are important for T2DM management.	252 (97.3)	0.97 ± 0.16
Total	1,008 (96.9)	3.88 ± 0.18

### Patients' diabetes-related practice

The practices of the patients with T2DM were assessed using 12 questions. Of the respondents, 54.1% participated in diabetes education activities in the past year, 54.5% followed dietary advice, 48.3% followed exercise advice, and 50.2% took medication as prescribed regularly in the past 6 months. The proportion of participants who checked blood pressure, blood sugar, and HbA1c was 35.1%, 32.8%, and 28.6%, respectively. Only 51.4% reported taking actions to prevent hypoglycemia during exercise. Of the respondents, 77.2% checked their feet in order to prevent diabetic foot complications, while only 23.6%, 24.7%, and 16.6% underwent lipid profile, renal function, and eye testing in the past year, respectively (Table 4).

**Table 4:** Description of patients’ practice scores in detail.

Item	Correct choice *n* (%)	Score (0–1) mean ± SD
P1. The frequency of participating in diabetes education activities in the past year	140 (54.1)	0.54 ± 0.50
P2. The adherence to a low-energy, low-fat as recommended in the past 6 months	141 (54.5)	0.57 ± 0.50
P3. The frequency of regular exercise in the past 6 months	125 (48.3)	0.48 ± 0.50
P4. The adherence to medication as prescribed regularly in the past 6 months	130 (50.2)	0.50 ± 0.50
P5. The frequency of blood pressure monitoring in the past 6 months	91 (35.1)	0.35 ± 0.48
P6. The frequency of blood sugar monitoring in the past 6 months	85 (32.8)	0.33 ± 0.47
P7. The frequency of HbA1c monitoring in the past 6 months	74 (28.6)	0.29 ± 0.45
P8. The measure taken to prevent hypoglycemia during exercise in the past 6 months	133 (51.4)	0.51 ± 0.50
P9. The frequency of foot examination in the past year	200 (77.2)	0.77 ± 0.42
P10. The frequency of blood lipid tests in the past year	61 (23.6)	0.24 ± 0.43
P11. The frequency of kidney function tests in the past year	64 (24.7)	0.25 ± 0.43
P12. The frequency of eye examination in the past year	43 (16.6)	0.17 ± 0.37
Total	1,287 (41.3)	4.96 ± 0.49

### Factors associated with patients' KAP

There were significant correlations between educational attainment, health insurance type, diabetic complications, and current medical treatment pattern with knowledge scores. Higher educational attainment was positively associated with better knowledge. Patients with health insurance and without complications had higher scores than their counterparts. Patients who did not receive any hypoglycemic medication had the lowest scores (12.20 ± 4.96), while patients took oral hypoglycemic medication had the highest scores (14.96 ± 4.39). Similarly, educational attainment, health insurance type, diabetic complications, and comorbidities influenced attitude scores. Patient with college or higher degrees had the highest scores (3.95 ± 0.28), while patients who were illiterate had the lowest scores (3.00 ± 1.70). Patients without complication or comorbidities scored higher than their counterparts, and patients with health insurance scored higher than those without. Educational attainment and marital status influenced the practice scores significantly. Overall, higher educational attainment had a positive impact on practice, and married participants had higher practice scores than single or divorced/widowed patients (Table 5).

**Table 5:** Associations of patients’ characteristics with KAP levels.

Variable	*n*	Knowledge scores		Attitude scores		Practice scores	
		Mean (SD)	*p*	Mean (SD)	*p*	Mean (SD)	*p*
Gender							
Male	144	13.13 ± 4.70	0.882	3.90 ± 0.54	0.697	5.10 ± 3.39	0.697
Female	115	13.03 ± 5.01		3.87 ± 0.54		4.81 ± 3.56	
Age							
<40	27	14.52 ± 4.68	0.109	4.00 ± 0.00	0.344	4.59 ± 4.61	0.402
40–59	157	13.21 ± 4.74		3.89 ± 0.47		5.20 ± 3.44	
≥60	75	12.31 ± 4.98		3.83 ± 0.72		4.61 ± 3.02	
Marital status							
Single	11	13.18 ± 5.40	0.405	4.00 ± 0.00	0.558	2.91 ± 3.56	0.023
Married	236	13.17 ± 4.80		3.87 ± 0.56		5.15 ± 3.47	
Divorced/widowed	12	11.25 ± 5.05		4.00 ± 0.00		3.25 ± 2.22	
Educational attainment							
Illiterate	10	9.50 ± 6.67	<0.001	3.00 ± 1.70	<0.001	5.10 ± 4.53	0.032
Primary and secondary school	137	11.75 ± 5.07		3.91 ± 0.46		4.38 ± 2.76	
High school	49	14.16 ± 3.78		3.89 ± 0.37		5.57 ± 3.32	
College and higher degrees	63	15.71 ± 3.03		3.95 ± 0.28		5.76 ± 4.46	
Health insurance type							
No	11	6.55 ± 5.47	<0.001	3.27 ± 1.35	<0.001	5.64 ± 3.91	0.515
Yes	248	13.38 ± 4.60		3.91 ± 0.46		4.94 ± 3.44	
Duration of diabetes mellitus							
<5 years	156	13.62 ± 5.00	0.091	3.94 ± 0.38	0.052	5.09 ± 3.71	0.781
5–9 years	79	12.22 ± 4.53		3.76 ± 0.79		4.76 ± 3.16	
≥10 years	24	12.50 ± 4.28		3.92 ± 0.28		4.88 ± 2.74	
Diabetic complications							
No	207	13.52 ± 4.76	0.004	3.94 ± 0.37	0.005	5.13 ± 3.61	0.135
Yes	52	11.37 ± 4.77		3.67 ± 0.92		4.33 ± 2.74	
Co-morbidities							
No	119	13.08 ± 4.94	0.408	3.94 ± 0.30	0.006	5.03 ± 3.58	0.309
Yes	140	13.09 ± 4.75		3.84 ± 0.67		4.92 ± 3.38	
Current medical treatment pattern							
Without medicine	137	12.20 ± 4.96	0.001	3.93 ± 0.48	0.242	5.67 ± 4.34	0.116
Oral medicine	73	14.96 ± 4.39		3.88 ± 0.50		4.51 ± 3.10	
Insulin	21	12.05 ± 3.93		3.67 ± 0.97		4.90 ± 2.39	
Oral medicine + insulin	28	13.29 ± 4.68		3.93 ± 0.38		5.43 ± 3.04	

## Discussion

### KAP among diabetic patients in rural areas in China

Using a self-administered questionnaire, we evaluated the baseline KAP level of patients with T2DM in Pingjiang County, a typical rural community in Hunan Province, Central South China. We found that our participants had good knowledge and positive attitudes regarding T2DM; however, substantial gaps were observed in their practices. Furthermore, we identified that educational attainment, health insurance type, diabetic complications, and current medical treatment pattern were significant predictors of knowledge; educational attainment, health insurance type, diabetic complications, and comorbidities were significant predictors of attitudes; and educational attainment and marital status significantly affected practices.

According to the study, knowledge scores were adequate, with overall values higher than 70 out 100, consistent with previous studies conducted in metropolitan Shanghai and other countries such as Ethiopia (21) and Iran (22). Contrary to our results, some studies reported poor diabetic knowledge among patients with diabetes (12, 14). Owning to the different instruments used and different ethnic or age groups, it is difficult to compare our results with those of other studies. However, our results demonstrate that the majority of our surveyed diabetic patients were familiar with basic aspects of diabetes, such as the definition of diabetes, risk factors, and signs and symptoms. This may be due to the efforts of the Chinese government to tackle diabetes, including the implementation of the National Basic Public Health Services (BPHS) program in 2009 (23) and national policies and programs to promote healthy lifestyles and prevent non-communicable diseases (24). We also observed that higher educational attainment had a positive impact on knowledge, consistent with findings from other studies (25–27). Patients with health insurance had higher scores than their counterparts, which echoed results from a study in Saudi Arabia (28), likely due to more access to medical resources for insured patients and more opportunities to receive education from their physicians. Meanwhile, patients without complications had higher scores than their counterparts. Furthermore, patients who did not receive any hypoglycemic medication had the lowest scores, while patients taking oral hypoglycemic medication had highest scores, a phenomenon also observed in other studies (29). Our study also indicated that the age and gender had no significant association with knowledge regarding diabetes, which was different from results in other developing countries (25, 27, 30) such as India, Saudi Arabia, and Bangladesh, possibly due to religious beliefs and specific sociocultural contexts.

Compared with studies from United Arab Emirates (11) and Benin (14), our study showed that the majority of respondents had positive attitudes toward diabetes. Most of the respondents viewed diabetes as a severe disease affecting almost every part of life, including risk of premature mortality and quality of life. Meanwhile, most participants thought all these adverse effects of diabetes could be eliminated by intensive blood glucose control. During the management of diabetes, most of them highlighted the importance of self-monitoring of blood glucose and diet or exercise. Our results were similar to those of another study conducted in United Arab Emirates, which reported a positive attitude among patients with diabetes (31). Similar to the findings for the knowledge subscale, we identified that educational attainment, health insurance type, comorbidities, and diabetic complications had significant influences on attitude scores.

Although our study indicated that most of the respondents possessed adequate knowledge and held positive attitudes regarding diabetes, these sentiments are not reflected in actual practice. Our results were consistent with those of other studies which reported poor practices among diabetic patients (12, 15, 22, 32). The overall score on practices was low. Importantly, our results indicated that greater knowledge or positive attitudes toward diabetes do not necessarily translate into better practices, a phenomenon observed in other studies as well (33, 34). This phenomenon may be partly due to the absence of obvious symptoms in patients with T2DM until advanced complications develop. Moreover, T2DM management is multifaceted, requiring structured education, self-management, and psychological support (35). These issues make T2DM management challenging and most patients can not adhere to the therapeutic regimen correctly. Consistent with other studies (21, 36), education attainment was significantly associated with practice. Our findings also indicated that marital status were significantly associated with practice, with married participants having higher practice scores than single or divorced/widowed patients. This maybe due to the fact that the practice of married patients were reminded and monitored by their spouses.

### General implications

The present study was conducted among rural diabetic patients in China to assess their KAP regarding diabetes. This study endeavors to help persons with diabetes in rural communities acquire the knowledge, self-care practices, and attitudes required for the effective management of diabetes, ultimately attenuating disparities in diabetes management for patients with T2DM in rural communities.

### Limitations

However, there are some limitations. First, this study was restricted to one geographic region in Central China due to which our findings cannot be generalized to all patients with diabetes in China. Second, this study was a cross-sectional study; therefore, only associations can be determined but not causal relationship. Third, the self-report survey may incur recall and social desirability biases, with more respondents reporting positive attitudes toward diabetes. Due to the limitations of the present study, further research is needed to confirm our findings and identify the most effective strategies for improving diabetes practices among Chinese patients.

## Conclusion

Despite adequate knowledge and positive attitudes toward diabetes, there were substantial gaps in diabetes-related practice. These findings call for urgent action from relevant health authorities and policymakers to improve diabetic practices among Chinese diabetic patients. In view of this, we make the following suggestions: (1) Regular education programs should be conducted by local health authorities. (2) More financial incentives should be provided to local primary care physicians in order to enhance the management of diabetes.

## Data Availability

The raw data supporting the conclusions of this article will be made available by the authors, without undue reservation.

## References

[1] International Diabetes Federation Brussels: IDF Diabetes Atlas. 10th edn (2021). Available online at: https://diabetesatlas.org (Accessed 25 October 2024).

[2] Li Y Teng D Shi X Qin G Qin Y Quan H Prevalence of diabetes recorded in mainland China using 2018 diagnostic criteria from the American diabetes association: national cross sectional study. Br Med J. (2020) 369:m997. 10.1136/bmj.m997 32345662 PMC7186854

[3] Zhong VW Yu D Zhao L Yang Y Li X Li Y Achievement of guideline-recommended targets in diabetes care in China: a nationwide cross-sectional study. Ann Intern Med. (2023) 176(8):1037–46. 10.7326/M23-0442 37523704

[4] Yang W Lu J Weng J Jia W Ji L Xiao J Prevalence of diabetes among men and women in China. N Engl J Med. (2010) 362(12):1090–101. 10.1056/NEJMoa0908292 20335585

[5] Xu Y Wang L He J Bi Y Li M Wang T Prevalence and control of diabetes in Chinese adults. Jama. (2013) 310(9):948–59. 10.1001/jama.2013.168118 24002281

[6] Wei Y Chen Y Zhao Y Rothman R Ming J Wang L Health literacy and exercise interventions on clinical outcomes in Chinese patients with diabetes: a propensity score-matched comparison. BMJ Open Diabetes Res Care. (2020) 8(1). 10.1136/bmjdrc-2020-001179 PMC7264995 32487594

[7] Ong-Artborirak P Seangpraw K Boonyathee S Auttama N Winaiprasert P. Health literacy, self-efficacy, self-care behaviors, and glycemic control among older adults with type 2 diabetes mellitus: a cross-sectional study in Thai communities. BMC Geriatr. (2023) 23(1):297. 10.1186/s12877-023-04010-0 37193967 PMC10185940

[8] Sweileh WM Zyoud SH Abu Nab'a RJ Deleq MI Enaia MI Nassar SM Influence of patients’ disease knowledge and beliefs about medicines on medication adherence: findings from a cross-sectional survey among patients with type 2 diabetes mellitus in palestine. BMC Public Health. (2014) 14(94). 10.1186/1471-2458-14-94 PMC3909379 24479638

[9] Gatt S Sammut R. An exploratory study of predictors of self-care behaviour in persons with type 2 diabetes. Int J Nurs Stud. (2008) 45(10):1525–33. 10.1016/j.ijnurstu.2008.02.006 18439609

[10] Plotnikoff RC Lippke S Courneya K Birkett N Sigal R. Physical activity and diabetes: an application of the theory of planned behaviour to explain physical activity for type 1 and type 2 diabetes in an adult population sample. Psychol Health. (2010) 25(1):7–23. 10.1080/08870440802160984 20391204

[11] Abdulrahman M Husain ZSM Abdouli KA Kazim MN Sayed Mahdi Ahmad F Carrick FR. Association between knowledge, awareness, and practice of patients with type 2 diabetes with socio-economic status, adherence to medication and disease complications. Diabetes Res Clin Pract. (2020) 163:108124. 10.1016/j.diabres.2020.108124 32259614

[12] Abbasi YF See OG Ping NY Balasubramanian GP Hoon YC Paruchuri S. Diabetes knowledge, attitude, and practice among type 2 diabetes mellitus patients in kuala muda district, Malaysia – A cross-sectional study. Diabetes Metab Syndr. (2018) 12(6):1057–63. 10.1016/j.dsx.2018.06.025 30017505

[13] Nguyen AT Pham HQ Nguyen TX Nguyen TTH Nguyen HTT Nguyen TN Knowledge, attitude and practice of elderly outpatients with type 2 diabetes Mellitus in national geriatric hospital, Vietnam. Diabetes Metab Syndr Obes. (2020) 13:3909–17. 10.2147/DMSO.S267866 33116737 PMC7588265

[14] Alaofè H Hounkpatin WA Djrolo F Ehiri J Rosales C. Knowledge, attitude, practice and associated factors among patients with type 2 diabetes in cotonou, southern Benin. BMC Public Health. (2021) 21(1):339. 10.1186/s12889-021-10289-8 33579243 PMC7881446

[15] Yang H Gao J Ren L Li S Chen Z Huang J Association between knowledge-attitude-practices and control of blood glucose, blood pressure, and blood lipids in patients with type 2 diabetes in Shanghai, China: a cross-sectional study. J Diabetes Res. (2017) 2017:3901392. 10.1155/2017/3901392 29464182 PMC5804409

[16] Strasser R Kam SM Regalado SM. Rural health care access and policy in developing countries. Annu Rev Public Health. (2016) 37:395–412. 10.1146/annurev-publhealth-032315-021507 26735432

[17] Jian W Chan KY Reidpath DD Xu L. China’s rural-urban care gap shrank for chronic disease patients, but inequities persist. Health Aff (Millwood). (2010) 29(12):2189–96. 10.1377/hlthaff.2009.0989 21134919

[18] Yang H Zhang S Zhu S Pan Z Yuan YZ Li S Evaluation of reliability and validity of Chinese version of diabetes, hypertension and hyperlipidemia(DHL) knowledge instrument in community diabetic patients. Br Med J. (2017) 16:277–82.

[19] Hu BB. Revision and Preliminary Application of the Chinese Version for Diabetes Empowerment Scale-Short Form. Beijing: Medical School of Zhejiang University, China (2010).

[20] Anderson RM Fitzgerald JT Gruppen LD Funnell MM Oh MS. The diabetes empowerment scale-short form (DES-SF). Diabetes Care. (2003) 26(5):1641–2. 10.2337/diacare.26.5.1641-a 12716841

[21] Asmelash D Abdu N Tefera S Baynes HW Derbew C. Knowledge, attitude, and practice towards glycemic control and its associated factors among diabetes Mellitus patients. J Diabetes Res. (2019) 2019:2593684. 10.1155/2019/2593684 31089472 PMC6476031

[22] Niroomand M Ghasemi SN Karimi-Sari H Kazempour-Ardebili S Amiri P Khosravi MH. Diabetes knowledge, attitude and practice (KAP) study among Iranian in-patients with type-2 diabetes: a cross-sectional study. Diabetes Metab Syndr. (2016) 10(1 Suppl 1):S114–9. 10.1016/j.dsx.2015.10.006 26610404

[23] National Health and Family Planning Commission. Notice of the National Health and Family Planning Commission on Printing and Distributing the National Basic Public Health Service Standard (Third Edition) China. Beijing: National Health and Family Planning Commission, PRC (2017).

[24] Wang H Zhai F. Programme and policy options for preventing obesity in China. Obes Rev. (2013) 14 Suppl 2(0 2):134–40. 10.1111/obr.12106 24102781 PMC4048452

[25] Shah VN Kamdar PK Shah N. Assessing the knowledge, attitudes and practice of type 2 diabetes among patients of saurashtra region, gujarat. Int J Diabetes Dev Ctries. (2009) 29(3):118–22. 10.4103/0973-3930.54288 20165648 PMC2822215

[26] Herath HMM Weerasinghe NP Dias H Weerarathna TP. Knowledge, attitude and practice related to diabetes mellitus among the general public in galle district in southern Sri Lanka: a pilot study. BMC Public Health. (2017) 17(1):535. 10.1186/s12889-017-4459-5 28571566 PMC5455097

[27] Saleh F Mumu SJ Ara F Begum HA Ali L. Knowledge and self-care practices regarding diabetes among newly diagnosed type 2 diabetics in Bangladesh: a cross-sectional study. BMC Public Health. (2012) 12:1112. 10.1186/1471-2458-12-1112 23267675 PMC3552981

[28] Mansy W Wajid S Alwhaibi A Alghadeer SM Alhossan A Babelghaith S Assessing Outpatients’ knowledge, attitude, and practice toward managing diabetes in Saudi Arabia. Inquiry. (2022) 59:469580221082781. 10.1177/00469580221082781 35377247 PMC8984850

[29] Abouammoh NA Alshamrani MA. Knowledge about diabetes and glycemic control among diabetic patients in Saudi Arabia. J Diabetes Res. (2020) 2020:1239735. 10.1155/2020/1239735 32215269 PMC7081031

[30] Islam FM Chakrabarti R Dirani M Islam MT Ormsby G Wahab M Knowledge, attitudes and practice of diabetes in rural Bangladesh: the Bangladesh population based diabetes and eye study (BPDES). PLoS One. (2014) 9(10):e110368. 10.1371/journal.pone.0110368 25313643 PMC4196995

[31] Al-Maskari F El-Sadig M Al-Kaabi JM Afandi B Nagelkerke N Yeatts KB. Knowledge, attitude and practices of diabetic patients in the United Arab Emirates. PLoS One. (2013) 8(1):e52857. 10.1371/journal.pone.0052857 23341913 PMC3544806

[32] Ardeňa GJ Paz-Pacheco E Jimeno CA Lantion-Ang FL Paterno E Juban N. Knowledge, attitudes and practices of persons with type 2 diabetes in a rural community: phase I of the community-based diabetes self-management education (DSME) program in san juan, batangas, Philippines. Diabetes Res Clin Pract. (2010) 90(2):160–6. 10.1016/j.diabres.2010.08.003 20828851

[33] Yezli S Yassin Y Mushi A Maashi F Aljabri N Mohamed G Knowledge, attitude and practice (KAP) survey regarding antibiotic use among pilgrims attending the 2015 Hajj mass gathering. Travel Med Infect Dis. (2019) 28:52–8. 10.1016/j.tmaid.2018.08.004 30118860

[34] Kasemy ZA Bahbah WA Zewain SK Haggag MG Alkalash SH Zahran E Knowledge, attitude and practice toward COVID-19 among Egyptians. J Epidemiol Glob Health. (2020) 10(4):378–85. 10.2991/jegh.k.200909.001 33009730 PMC7758851

[35] Chatterjee S Khunti K Davies MJ. Type 2 diabetes. Lancet. (2017) 389(10085):2239–51. 10.1016/S0140-6736(17)30058-2 28190580

[36] Niguse H Belay G Fisseha G Desale T Gebremedhn G. Self-care related knowledge, attitude, practice and associated factors among patients with diabetes in ayder comprehensive specialized hospital, north Ethiopia. BMC Res Notes. (2019) 12(1):34. 10.1186/s13104-019-4072-z 30658687 PMC6339268

